# Bidirectional Mendelian randomization and cross-sectional study reveal depression as a causal risk factor for endometriosis

**DOI:** 10.1097/MD.0000000000045729

**Published:** 2025-10-31

**Authors:** Liangzi Jiang, Lingling Jiang, Suting Ma

**Affiliations:** a Department of Obstetrics and Gynecology, Shandong Provincial Qianfoshan Hospital, Key Laboratory of Laparoscopic Technology, the First Affiliated Hospital of Shandong First Medical University, Jinan, China; b Department of Gastroenterology, Linyi Geriatric Hospital, Linyi, China; c Department of Pediatrics, Linyi People’s Hospital, Linyi, China.

**Keywords:** depression, endometriosis, Mendelian randomization, NHANES

## Abstract

Endometriosis (EMs) and depression are both common conditions that have a significant negative impact on quality of life. However, it is still unclear about the relationship between them. The study was designed to investigate the phenotypic and causal association between depression and EMs. For its nationally representative sample, comprehensive health data and publicly accessibility, we chose data from the National Health and Nutrition Examination Survey for our observational study section including 1393 participants in 2005 to 2006 year cycle which contains information both on depression assessed by the Patient Health Questionnaire 9 and self-reported EMs. Initially, we conducted logistic regression analysis to evaluate phenotypic association between depression and EMs utilizing extensive data taken from National Health and Nutrition Examination Survey. Then, using data from the genome-wide association study (female self-reported depression data: N = 194,153 and female medical-recorded depression data: N = 192,680 from UK Biobank where sex-stratified data was provided; EMs data: N = 150,350 from FinnGen consortium database), we assessed the genetically predicted association performing bidirectional two-sample Mendelian randomization (MR) analysis. Inverse-variance weighted method was employed as the main MR analysis. The Weighted median, MR Egger, Simple mode, Weighted mode, and MR pleiotropy residual sum and outlier test were also performed to assess the robustness of our findings. EMs was present in almost 13.64% of the participants in the depression group, which was much greater than the 6.63% in the non-depression group. Depression and EMs were positively correlated in the observational analysis (OR = 2.44, 95%CI = 1.26–4.74). Furthermore, each greater Patient Health Questionnaire 9 score was associated with a 7% increased risk of EMs. EMs did not have a genetically informed influence on depression, while depression clearly had a genetically predicted effect on EMs, according to bidirectional MR analysis. This study shows a genetically predicted association between depression and EMs as well as a favorable phenotypic correlation. It implies that monitoring of diseases of the reproductive system, such as EMs, in female patients with mood disorders and treating them from a multidisciplinary perspective should not be neglected. Further large-scale prospective cohort studies or mechanistic research are warranted to confirm these results and explore the underlying biological mechanisms.

## 1. Introduction

Endometriosis (EMs) is known as the growth and accumulation of endometrial tissue outside the uterine cavity, which typically lines the lining of the uterus.^[1–3]^ Roughly 10% of women worldwide at the reproductive age suffer with this prevalent and complicated illness. While EMs is believed to be caused by a variety of variables, the exact etiology and pathophysiology of EMs remain unclear.^[4,5]^ According to epidemiological research, EMs patients frequently experience mood disorders like depression and anxiety, which have a major negative impact on their mental health.^[6,7]^

Depression is a prevalent mental health disorder and has steadily developed into a study hotspot for the carcinogenesis and progression of EMs. Individuals with depression have shown severe symptoms and appeared to be more susceptible to EMs.^[6,8]^ EMs and depression may have similar inflammatory pathways and immune system dysfunction. The direct relationship between depression and EMs is still unknown, despite the fact that numerous observational studies have found an association between the 2 conditions.^[9,10]^ However, the results of observational studies are susceptible to various bias and mixed by confounders, leading to erroneous or exaggerated results.

Numerous genetic variants linked to complex traits have been found through genome-wide association studies (GWAS), which are also frequently utilized to supply the best instrumental variants (IVs) for Mendelian randomization (MR) analysis. Since genetic variants are presumed to inherit randomly, and alleles are not influenced by diseases, the MR technique has been frequently utilized to minimize the influence of biases, reverse causes, and confounders.^[11,12]^ MR investigates the genetically predicted effects of exposure on outcome using genetic variants linked to exposure. In order to investigate the genetical relationships between depression and EMs, we conducted bidirectional two-sample MR analyses in this study.

## 2. Materials and methods

### 2.1. National Health and Nutrition Examination Survey (NHANES) study design and participants

**Figure 1.**
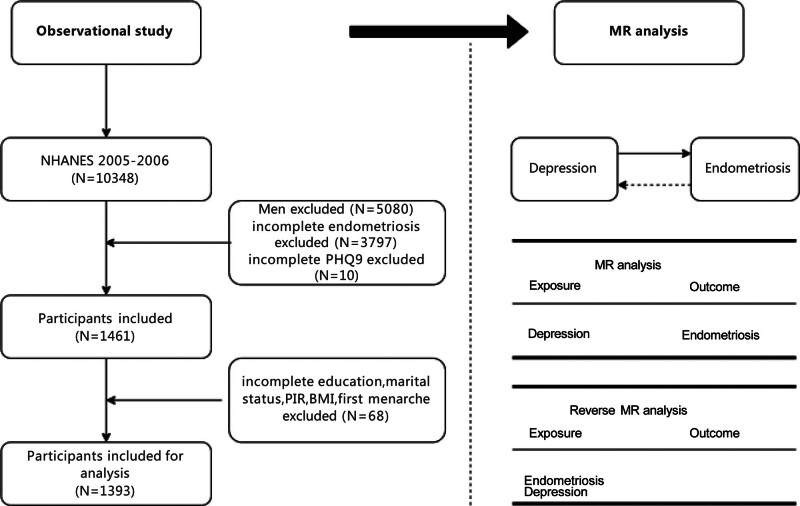
The flowchart of participants selection in NHANES and Mendelian randomization study design. BMI = body mass index, MR = Mendelian randomization, NHANES = National Health and Nutrition Examination Survey, PHQ9 = Patient Health Questionnaire, PIR = poverty income ratio.

NHANES is a nationally representative survey conducted by the National Center for Health Statistics in which stratified, multistage probability cluster sampling has been used to assess the health or nutritional status of the noninstitutionalized US population. The NHANES protocol was approved by the review board of National Center for Health Statistics and all data were collected with participants’ written consent. we used only de-identified data for secondary analysis, so ethical approval was waived in this analysis. Because of its nationally representative sample, comprehensive health data and publicly accessibility, we chose data from NHANES for our study. Information both on Patient Health Questionnaire 9 (PHQ9) and EMs was only provided in the NHANES 2005–2006 cycle. Only women between the ages of 20 and 54 in this year cycle were included, while men were not. At the same time, those who had insufficient data on confounders, the PHQ9, and self-reported EMs were eliminated. The final association analysis included 1393 participants, as Figure 1 illustrates.

### 2.2. Definition of EMs and depression in NHANES

Based on acceptable diagnostic criteria for depression, the PHQ9 is a valid tool for assessing mood.^[13,14]^ Nine questions on the PHQ9 scale are evaluated from “0” to “3”, and the overall score ranges from “0” to “27.”^[15]^ PHQ9, which was given in a Mobile Examination Center (MEC), was used to assess the survey respondents’ levels of depression.^[16]^ Depression was defined as a total PHQ9 score of ≥ 10.^[17]^

The “rhq360” questionnaire applied in the MEC was used to determine EMs. “Has a doctor or other health professional ever told you that you have endometriosis?” was one of the questions on the structured questionnaire.^[18]^ Those who selected “yes” were placed in the case group, which included people who self-reported having EMs, and those who selected “no” were placed in the control group, which did not.

### 2.3. Other covariates

Home interviews were used to gather demographic factors such as age, race/ethnicity, marital status, education, and family income. Mexican American, non-Hispanic White, non-Hispanic Black, and other races were the categories of race and ethnicity. Married/living with a partner, or not married were the 3 categories of marital status. The participants’ educational background was noted as either less than high school, high school graduate, or some college or higher. According to the poverty income ratio (PIR), family income was assessed and categorized as low (PIR < 1.35), medium (1.35 ≤ PIR < 3.0), and high (PIR ≥ 3.0).^[19]^ The MEC assessed health-related factors such as alcohol consumption, diabetes, hypertension, and smoking status. The question “smoked at least 100 cigarettes in life?” was used to characterize smoking. The response “had at least 12 alcohol drinks/one year or lifetime” was used to determine alcohol intake. The response to the question, “Doctor told you have diabetes,” was used to define diabetes. The response to the question “ever told you had high blood pressure” was used to define hypertension. Using “rhq” 010, 131, and 420 questionnaires, age at menarche, history of pregnancy, and usage of oral contraceptives were included as disease-related factors.^[20–22]^

### 2.4. Mendelian randomize studies

The bidirectional two-sample MR analysis was used to evaluate the causal link between EMs and depression, as simply illustrated in Figure 1. Data for depression were extracted summary GWAS data from UK Biobank which provided sex-stratified data, including a total of 194,153 female individuals with self-reported depression analyzing 13,791,461 variants for primary analysis, and a total of 192,680 female individuals seen a psychiatrist for nerves anxiety tension or depression named medical-recorded depression analyzing 13,791,468 variants for replication analysis. FinnGen (https://r12.finngen.fi/) provided the summary GWAS data for EMs, which includes 20,190 cases and 130,160 controls with 21,303,852 variations analyzed. The FinnGen dataset’s EMs diagnosis should satisfy the matching ICD-9 or ICD-10 criteria. The FinnGen sample’s median age was around 37.27 years old. No overlap existed between exposure and outcome samples. Racial stratification bias was reduced because all subjects were European. As these data are fully anonymized aggregate results with no individual-level genotype or phenotype information, ethical approval was not necessary for this research. Table S1, Supplemental Digital Content, https://links.lww.com/MD/Q546 lists the specifics of the GWAS data used in this MR analysis.

### 2.5. IVs selection

For a bidirectional two-sample MR analysis, in order to ensure the validity of causal inferences, the selected instrumental variables (IVs) must satisfy 3 key assumptions: they must be strongly associated with the exposure (relevance assumption); they must be independent of confounding factors (independence assumption); and they must influence the outcome solely through the exposure (exclusion-restriction assumption).^[23]^ In order to prioritize genetic variants that met the 3 instrumental assumptions for MR analysis, we used a methodical process. First, using a modified significance criterion (*P* < 5 × 10^−6^), single-nucleotide polymorphisms (SNPs) linked to depression were retrieved as IVs. Since there were fewer instrumental variables available for MR analysis when using stricter criteria like *P* < 5 × 10^−8^ or *P* < 5 × 10^−7^, the barrier should be loosened to *P* < 5 × 10^−6^. Previous psychiatric MR investigations also embraced this change in the statistical threshold for genetic instrumentation.^[24,25]^ Second, SNPs in linkage disequilibrium were excluded (*r*^2^ < 0.001 within a window of 10,000 kb), and the remaining SNPs were extracted from the resulting dataset. Thirdly, to determine the percentage of exposure variance that the instrument could account for, *R*^2^ was computed. We used the formula *R*^2^ = 2 × EAF × (1 − EAF) × β,^[2]^ where EAF denotes the effect allele frequency of the SNP, and β indicates the estimated effect of the SNP on the trait. To assess the degree of correlation between IVs and exposure, the F-statistic was computed independently for each IV.^[26]^ The F-statistic was determined using the formula F = *R*^2^(N − 2)/(1 − *R*^2^), with N being the sample size of the GWAS related to the trait. Weak genetic tools (F-statistic < 10) were excluded to ensure the strength of the exposure. Consequently, self-reported depression and medical-recorded depression were found to be substantially correlated with 23 and 49 SNPs, respectively, as IVs. A more stringent threshold of *P* < 5 × 10^−8^ (linkage disequilibrium *r*^2^ < 0.001 within 10,000 kb) was established for strong association when considering EMs as exposure for reverse MR analysis. Consequently, 31 independent SNPs were chosen to be IVs. Following the removal of palindromic instrumental variations from harmonization of the exposure and outcome datasets, MR analysis was performed on the remaining IVs. A webtool (https://sb452.shinyapps.io/power/) was determined to estimate the post statistical power for binary outcomes set.

### 2.6. Statistical analysis

The observational analysis was first conducted based on NHANES data. Using the *t*-test for continuous variables and the chi-square test for categorical data, baseline was analyzed to evaluate the various features based on depression status. To investigate the relationship between depression and EMs, multivariate logistic regression analyses were conducted adjusting for different potential confounders. Confounders were chosen when in univariate analysis, their *P* values were <.05 (Table S2, Supplemental Digital Content, https://links.lww.com/MD/Q546) or based on previous findings.^[22,27–32]^ Model 1 was adjusted for sociodemographic variables (age, race/ethnicity, education, marital status, and family income). Model 2 was added lifestyle and related disease variables (body mass index [BMI], alcohol, smoking status, hypertension, and diabetes) based on model 1. Model 3 was added reproductive variables (age at menarche, pregnant history, and oral contraceptive use) based on model 2. To better understand these findings, subgroup analyses were performed by age (<40, ≥40), Marital status, Race/ethnicity, education, family income, and BMI (<25, ≥25).

The MR analysis was initially performed using random-effect inverse-variance weighted (IVW) approach by pooling the Wald ratio for IVs to explore to assess the causality of depression and EMs in both primary analysis and replication analysis.^[33]^ In order to compare the results of the IVW approach with the possible cause effects when IVs deviated from standard assumptions, a number of other well-established robust sensitivity techniques were employed, including as the Weighted median,^[34]^ MR Egger,^[35]^ Simple mode,^[36]^ and Weighted mode methods.^[37]^ The effect estimates of each IV on exposure and outcome were displayed through scatter plots. Directional pleiotropy was detected via the MR-Egger regression’s intercept and MR pleiotropy residual sum and outlier (MR-PRESSO) global test.^[38]^ MR-PRESSO analysis was used to identify and remove significant outlier SNPs that may introduce horizontal pleiotropy. To evaluate heterogeneity, a funnel plot was created, and the Cochran *Q*-test was run. Additionally, the leave-one-out analysis was used to determine whether a single SNP was responsible for the entire effect in order to assess the stability of the results. The statistical significance criteria were set at *P* < .05 and R software (version 4.4.1; The R Foundation for Statistical Computing, Vienna, Austria) with TwoSampleMR package and Free statistical software (version 1.9; Free Clinical Medical Technology Co., Ltd., Beijing, China) were employed for statistical analyses.

## 3. Results

### 3.1. Baseline characteristics of subjects from NHANES

Table 1 indicates that 13.64% of patients in the depression group had EMs, considerably greater than that of the normal group (6.63%). Patients with and without depression showed differences significantly (*P* < .05) in age, race/ethnicity, education, family income, BMI, smoking status, hypertension, history of pregnancy, and PHQ9 score.

**Table 1:** Baseline characteristics of the participants with and without depression.

Characteristic	Total	Depression		*P*-value
		No	Yes	
	n = 1393	n = 1283	n = 110	
Age (yr), Mean ± SD	35.17 ± 10.03	34.96 ± 9.96	37.63 ± 10.55	.007
Race/ethnicity, n (%)				.019
Mexican American	302 (21.68)	281 (21.9)	21 (19.09)	
Non-Hispanic White	643 (46.16)	603 (47)	40 (36.36)	
Non-Hispanic Black	319 (22.90)	281 (21.9)	38 (34.55)	
Other Race	129 (9.26)	118 (9.2)	11 (10)	
Marital status, n (%)				.117
Married or living with a partner	930 (66.76)	864 (67.34)	66 (60)	
Not married	463 (33.24)	419 (32.66)	44 (40)	
Education, n (%)				.006
Less than high school	287 (20.60)	254 (19.8)	33 (30)	
High school graduate	291 (20.89)	263 (20.5)	28 (25.45)	
Some college or above	815 (58.51)	766 (59.7)	49 (44.55)	
Family income, n (%)				<.001
Low	375 (26.92)	321 (25.02)	54 (49.09)	
Medium	511 (36.68)	470 (36.63)	41 (37.27)	
High	507 (36.40)	492 (38.35)	15 (13.64)	
BMI, Mean ± SD	28.97 ± 7.51	28.82 ± 7.34	30.67 ± 9.18	.013
Alcohol, n (%)				.225
Yes	848 (60.88)	787 (61.34)	61 (55.45)	
No	545 (39.12)	496 (38.66)	49 (44.55)	
Smoking, n (%)				<.001
Yes	511 (36.68)	450 (35.07)	61 (55.45)	
No	882 (63.32)	833 (64.93)	49 (44.55)	
Diabetes, n (%)				.14
Yes	61 (4.38)	53 (4.13)	8 (7.27)	
No	1332 (95.62)	1230 (95.87)	102 (92.73)	
Hypertension, n (%)				.007
Yes	249 (17.88)	219 (17.07)	30 (27.27)	
No	1144 (82.12)	1064 (82.93)	80 (72.73)	
Age at menarche, n (%)				.595
<13	726 (52.12)	666 (51.91)	60 (54.55)	
≥13	667 (47.88)	617 (48.09)	50 (45.45)	
Pregnant history, n (%)				.003
Yes	1178 (84.57)	1074 (83.71)	104 (94.55)	
No	215 (15.43)	209 (16.29)	6 (5.45)	
Oral contraceptive, n (%)				.656
Yes	1078 (77.39)	991 (77.24)	87 (79.09)	
No	315 (22.61)	292 (22.76)	23 (20.91)	
PHQ9, Mean ± SD	3.26 ± 4.18	2.30 ± 2.42	14.39 ± 4.29	<.001
Endometriosis, n (%)				.006
No	1293 (92.82)	1198 (93.37)	95 (86.36)	
Yes	100 (7.18)	85 (6.63)	15 (13.64)	

Classification of depression: no (total PHQ9 score < 10), yes (total PHQ9 score ≥ 10).

BMI = body mass index, PHQ = Patient Health Questionnaire.

### 3.2. The relationship between depression and EMs

**Figure 2.**
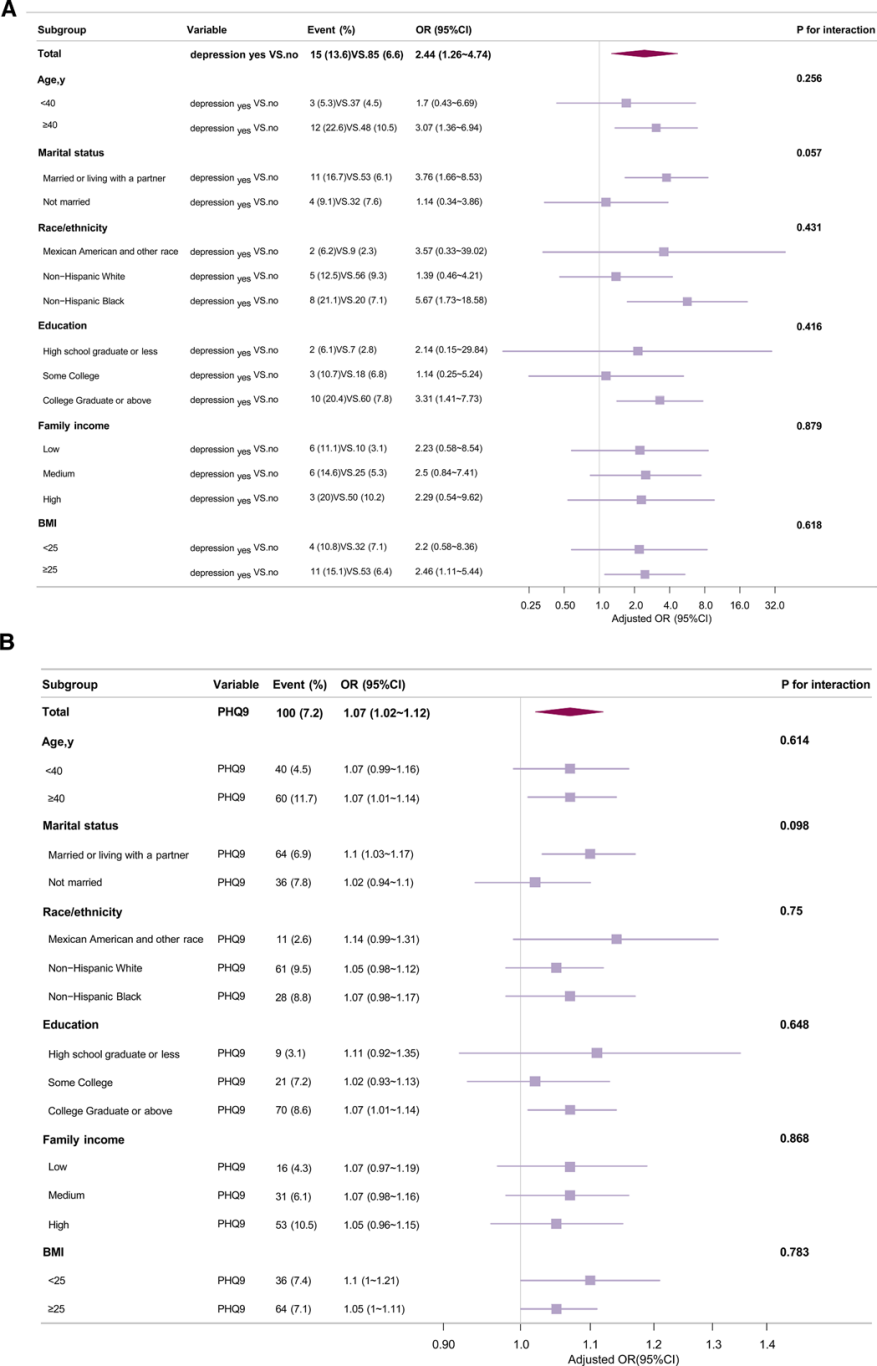
Subgroup analyses of depression (A) and PHQ9 score (B) on the prevalence of endometriosis. BMI = body mass index, CI = confidence interval, OR = odds ratio, PHQ9 = Patient Health Questionnaire.

Depression and EMs were found to be positively correlated by logistic regression analyses in 4 different models after adjusting various confounders. In the crude model the odds ratio (OR) of EMs was 2.23 (95% CI: 1.24–4.0, *P* = .015). In the fully adjusted model 3 the OR was 2.44 (95% CI: 1.26–4.74, *P* = .007), as indicated in Table 2. Additionally, the results showed a favorable correlation between the PHQ9 score on EMs. According to the crude model, participants with an elevated PHQ9 score had a 5% greater probability of developing EMs. The probability increases to 7% in the fully adjusted model 3. The results were consisted in subgroup analysis and no significant interactions were found between covariates and depression (*P* for interaction > .05; Fig. 2).

**Table 2:** Association between PHQ9, depression and odds of endometriosis.

Variables	OR (95%CI)							
	Crude	*P*-value	Model 1	*P*-value	Model 2	*P*-value	Model 3	*P*-value
PHQ9	1.05 (1.01–1.09)	.015	1.06 (1.01–1.11)	.009	1.06 (1.02–1.11)	.007	1.07 (1.02–1.12)	.007
Depression								
No	1 (Ref)		1 (Ref)		1 (Ref)		1 (Ref)	
Yes	2.23 (1.24–4.0)	.008	2.43 (1.28–4.62)	.007	2.51 (1.29–4.85)	.006	2.44 (1.26–4.74)	.008

Model 1 was adjusted for sociodemographic variables (age, race/ethnicity, education, marital status, and family income). Model 2 was adjusted for Model 1 + lifestyle and related disease variables (BMI, alcohol, smoking status, hypertension, and diabetes). Model 3 was adjusted for Model 2 + reproductive variables (age at menarche, pregnant history, and oral contraceptive use).

CI = confidence interval, OR = odds ratio, PHQ = Patient Health Questionnaire, Ref = reference.

### 3.3. MR analysis of depression on EMs

**Figure 3.**
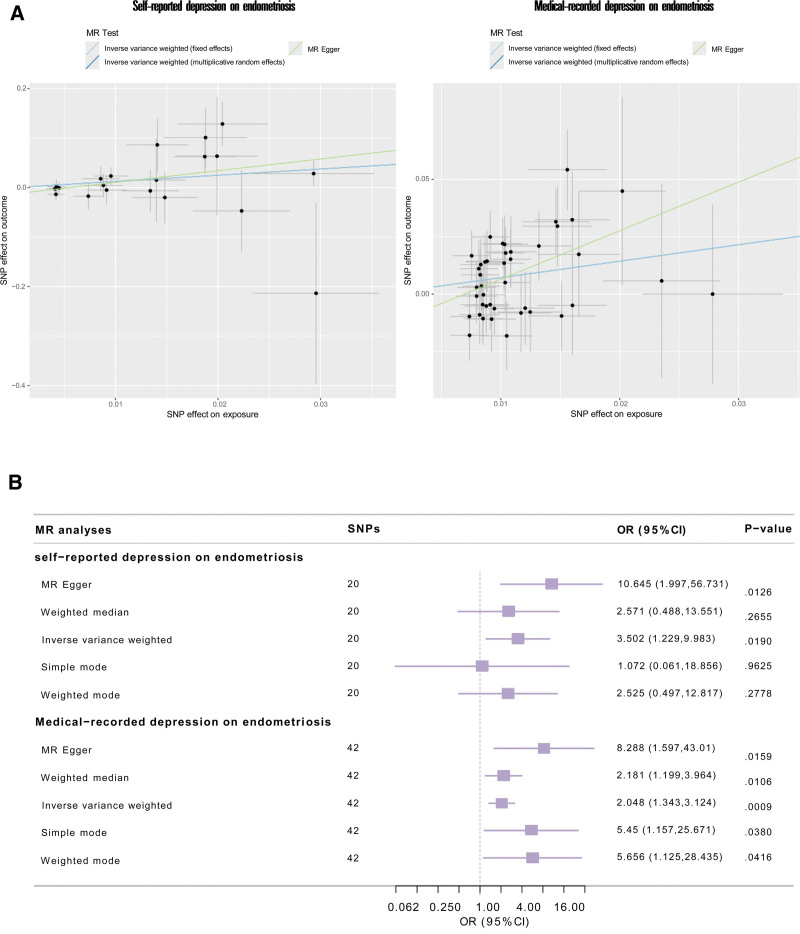
Mendelian randomization estimates of the association between self-reported depression and medical-recorded depression on endometriosis. (A) The scatter plots of MR analyses. (B) The forest plot showing the results of each MR estimates. CI = confidence interval, MR = Mendelian randomization, OR = odds ratio, SNP = single-nucleotide polymorphism.

**Figure 4.**
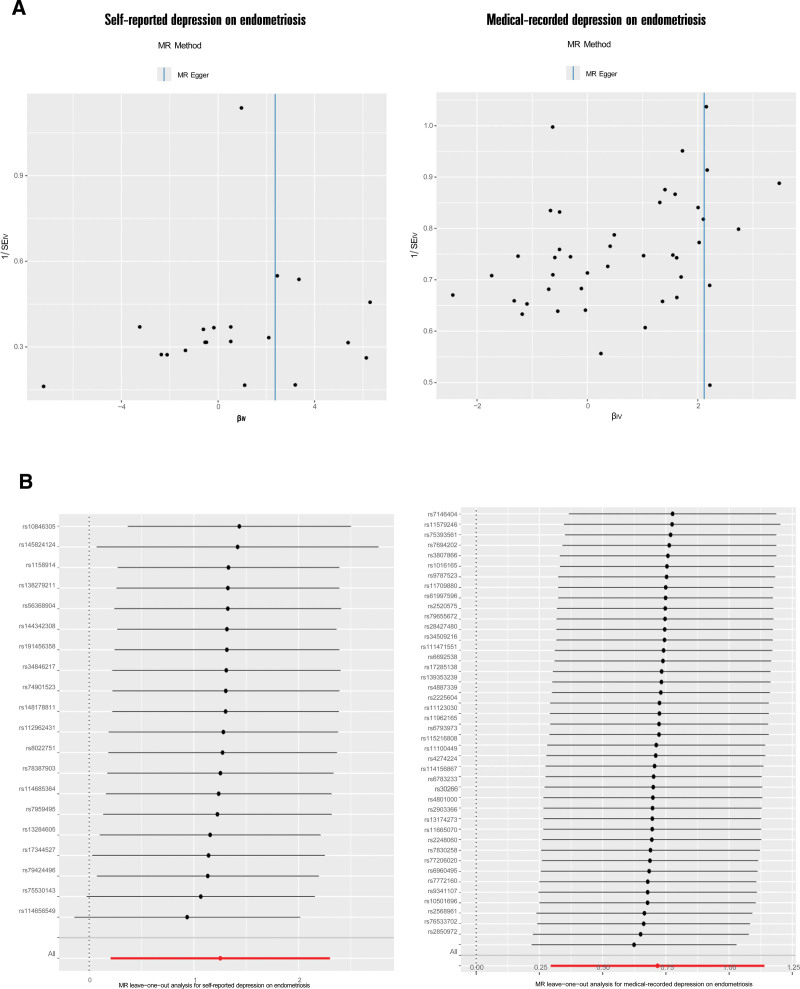
Heterogeneity and robustness assessment of MR analyses. (A) The funnel plots for assessing heterogeneity. (B) The leave-one-out plots for assessing the robustness. MR = Mendelian randomization.

In the primary analysis, 23 SNPs genetically linked to self-reported depression were selected as IVs with F-values ranged from 21.449 to 38.043. After eliminating 3 absent from the EMs summary GWAS dataset, 20 IVs for self-reported depression were obtained for the MR analysis. Details are showing in Table S3, Supplemental Digital Content, https://links.lww.com/MD/Q546. According to the IVW model, self-reported depression significantly contributed to EMs (OR = 3.502 [1.229–9.983], *P* = .019), as illustrated in Figure 3. The MR-Egger model yielded a similar estimate (OR = 10.645 [1.997–56.731], *P* = .0126). Figure 3 displayed the forest plot and scatter plot. The MR-Egger regression intercept, which showed no pleiotropy, was −0.013 (*P* = .1129). MR-PRESSO Global test also found no horizontal pleiotropy (*P* = .479). There was no heterogeneity according to the Cochran *Q* test (*P* = .5678) as demonstrated by the funnel plot’s symmetry (Fig. 4A). Furthermore, no single IV significantly impacted the genetic relationship between self-reported depression and EMs, according to the leave-one-out analysis (Fig. 4B).

In the replication analysis, 42 of 49 IVs were chosen represented for medical-recorded depression with F-values ranged from 20.891 to 42.951 after eliminating 3 absent from the EMs summary GWAS dataset and 4 palindromic IVs (Table S3, Supplemental Digital Content, https://links.lww.com/MD/Q546). Medical-recorded depression was associated with EMs (OR = 2.048 [1.343–3.124], *P* = .0009) in the IVW model. The MR-Egger model, weighted median model, simple mode and weighted mode yielded similar estimates. Figure 3 displayed the forest plot and scatter plot of the results. There was neither pleiotropy in MR-Egger regression with an intercept of −0.015 (*P* = .0934) nor in MR-PRESSO Global test (*P* = .363; Table S5, Supplemental Digital Content, https://links.lww.com/MD/Q546). No heterogeneity was found according to the Cochran *Q* test (*P* = .347) as demonstrated by the funnel plot’s symmetry (Fig. 4A). Furthermore, in the leave-one-out analysis, no significant effect changed after sequentially removing IVs (Fig. 4B). This genetic association is well supported by the 100% post statistical power.

### 3.4. Reverse MR analysis of EMs on depression

In the reverse analysis, among 31 SNPs with F-values ranged from 20.891 to 42.951, 29 IVs were selected for EMs to perform the MR analysis after removing 1 palindromic IV and 1 missing in both depressive summary GWAS datasets (Table S4, Supplemental Digital Content, https://links.lww.com/MD/Q546). The IVW model showed that self-reported depression was not substantially influenced by EMs (OR = 0.999 [0.996–1.003], *P* = .9968). Estimates using the weighted median model, MR-Egger model, simple mode, and weighted mode were comparable. There was no pleiotropy in the MR-Egger regression intercept, which was −1.156 × 10^−4^ (*P* = .8137), so was in MR-PRESSO Global test (*P* = .479). The Cochran *Q* test revealed no heterogeneity (*P* = .6485; Table S6, Supplemental Digital Content, https://links.lww.com/MD/Q546).

To verify the results in the replication cohort, we used medical-recorded depression as the outcome. No association was found between EMs and medical-recorded depression (OR = 1.006 [0.997–1.014], *P* = .2085). The Egger-intercept was −2.072 × 10^−3^ (*P* = .0857), indicating that no pleiotropy was observed. However, MR-PRESSO Global test (*P* = .024) showed that some pleiotropic effects existed. After the exclusion of 1 outlier (rs6938760) identified in MR-PRESSO test, there was no pleiotropy observed between EMs and medical-recorded depression. The distortion test *P* value was .252, indicating that the removal of the outlier did not significantly affect the genetic estimates. The Cochran *Q*-test indicated some heterogeneity (*P* = .0433; Table S6, Supplemental Digital Content, https://links.lww.com/MD/Q546). Considering that heterogeneity is acceptable in random-effects IVW method, the result remains reliable.

## 4. Discussion

Using NHANES data, we discovered that patients with depression had a higher rate of EMs. We also found that depression was an independent risk factor for EMs in both crude and multivariable regression models, and the results persisted in subgroup analysis. Then, using bidirectional two-sample MR, we investigated the genetically predicted connection between depression and EMs risk. A greater likelihood of EMs was linked to genetically predicted depression, and many MR techniques supported this finding. Conversely, EMs and depression did not appear to be genetically related, according to inverse MR analysis. All of these findings suggested that EMs is significantly influenced by depression.

We are interested in the co-occurrence of depression and EMs since depression is closely linked to gynecological disorders.^[39]^ Depression symptoms are far more common in EMs patients.^[6]^ The findings of the many epidemiological research that have investigated the relationship between depression and EMs remain mixed. For instance, mild depression was identified as a significant risk factor for EMs in cross-sectional research.^[9]^ However, a long-term follow-up investigation discovered that EMs was linked to a higher risk of depression.^[40]^ Furthermore, EMs was revealed to be bidirectionally associated with both anxiety and depression in a phenotype-based longitudinal study based on a large-scale Swedish study.^[10]^ These discrepancies in orientation might result from various participant scales and endpoint definitions. Therefore, we extracted information from 2005 to 2006 NHANES database, defined depression using PHQ-9. Consistent with some previous studies, this study found a positive association between depression and constipation.

However, because observational studies are unable to completely rule out the influence of potential confounders, they are unable to establish a causal relationship between outcome and exposure. By adding instrumental variables that meet the fundamental requirements, MR prevents reverse causation and enables the investigation of causality in epidemiological research.^[41]^ More GWAS studies have reported a significant causal relationship between depression and EMs. Dora Koller et al provided evidence of genetic associations of EMs with emotional disorders.^[42]^ But they used 1-sample MR analysis, which may lead to inaccurate causal associations due to sample selection bias, inability to exclude environmental confounders, etc. Another two independent MR analyses also found mood disorders were causal risk factors of EMs and adenomyosis.^[43,44]^ However, they both used genetic data unstratified by gender to represent female depression, which could induce false associations. Moreover, the latter study used genetic instrumental variables of exposure and outcome partial from a same GWAS database, inducing possible overlapping of samples. In this study, we extracted instrumental variables from 2 different databases represented two independent populations. More importantly, we used female-specific genetic IVs represent for depression from UK Biobank. Thus, we reached a more reliable conclusion that depression had a causal effect on EMs and vice versa. We did not find a reverse causality, possibly limited by the database, we could neither completely rule out the influence of residual confoundings or unknown genetic factors.

We still don’t know the precise mechanism underlying this causal effect. The substantial genetic overlap and association between depression and EMs may have been the cause of this.^[45]^ When examining the effect of depression on EMs, inflammatory markers should also be taken into account. Previous studies have shown that chronic inflammation and immune system irregularities are effective in the development of depression. In particular, proinflammatory cytokines and immunological changes may form a common biological basis in both depression and EMs patients. Recent studies have shown that immunological and inflammatory markers such as neutrophil gelatinase-associated lipocalin, Granzyme B and ADAMTS-1/ADAMTS-9 are found at abnormal levels in patients with EMs.^[46–48]^ Some scholars have found that depression and EMs share some other elevated levels of inflammatory biomarkers from genetic perspective and by basic experiment.^[49,50]^ A study by Cuevas et al revealed that stress may exacerbate EMs in animal models by producing inflammatory mediators such as IL-33,^[51]^ which means that inflammatory factors may play a mediating role in depression–EMs relationship. Dysregulation of the hypothalamic–pituitary–adrenal axis may be another explanation. Mokhtari et al indicated that chronic stress had been found to activate the hypothalamic–pituitary–adrenal axis, causing the dysregulation of corticotropin-releasing factor, leading to elevated glucocorticoid receptors in immune cells, which can bind to systemic cortisol and become activated.^[7]^ The immune imbalance may exacerbate the progression of EMs. At the meantime, elevated cortisol can variably raise Th2 cytokines by inhibiting the Th1 immune response. A similar shift in the immune response was seen in the ascites of patients with EMs.^[52]^ Though whether or not mental health interventions including depression management could reduce the risk of EMs is still unknown, they have been shown to control various symptoms and improve quality of life for people with EMs.^[53,54]^

There are some limitations in the current study. First, it is unclear whether our findings can be applied to other populations because the GWAS data used in this study came exclusively from European populations without considering the potential confounding impact of racial factors on the mediation of EMs. Second, we selected IVs for depression using a relaxed significance threshold of *P* < 5 × 10^−6^, which may have resulted in bias and false-positive variants. All IVs’ F-statistics, however, were >10, indicating a lower chance of weak instrument bias. Similarly, when using IVs for mood disorders, a number of additional research employed the same significant level (*P* < 5 × 10^−6^).^[24,55]^ Third, the lack of longitudinal follow-up in NHANES limits the reliability of the cross-sectional results. Fourth, this study did not examine the precise mechanism by which EMs is caused by depression-related features. It is necessary to conducting large-scale prospective cohort studies or mechanistic research to clarify the precise pathophysiological mechanism.

## 5. Conclusions

This is the 1st study using data from NHANES combing GWAS with gender stratified IVs to reveal a positive phenotypic association and a causal effect of depression on EMs. Further large-scale prospective cohort studies or mechanistic research are warranted to confirm these results and clarify the precise pathophysiological mechanism. In the meantime, monitoring of diseases of the reproductive system, such as EMs, in female patients with mood disorders should not be neglected. Though whether or not mental health interventions including depression management could reduce the risk of EMs is still unknown, they have been shown to control various symptoms and improve quality of life for people with EMs.

## Author contributions

**Conceptualization:** Liangzi Jiang, Lingling Jiang, Suting Ma.

**Data curation:** Liangzi Jiang, Lingling Jiang.

**Methodology:** Liangzi Jiang, Lingling Jiang, Suting Ma.

**Project administration:** Suting Ma.

**Supervision:** Suting Ma.

**Visualization:** Liangzi Jiang, Suting Ma.

**Writing – original draft:** Liangzi Jiang, Lingling Jiang.

**Writing – review & editing:** Liangzi Jiang, Lingling Jiang, Suting Ma.

## Supplementary Material


