# Supplementary material for: Bidirectional Mendelian randomization and cross-sectional study reveal depression as a causal risk factor for endometriosis
Source: Medicine (Baltimore). 2025 Oct 31;104(44):e45729. doi: 10.1097/MD.0000000000045729 (PMC12582783; doi:10.1097/MD.0000000000045729)
Supplement: Supplementary file 1 [file medi-104-e45729-s001.docx]

**Supplementary Table S1: Mendelian randomization analysis of tea intake**

| SNP | chr | position | Effect allele | Other allele | Beta | SE | P |
| --- | --- | --- | --- | --- | --- | --- | --- |
| rs11587444 | 1 | 150722844 | G | A | 0.0140328 | 0.00217078 | 1.00E-10 |
| rs11164870 | 1 | 93552187 | G | C | -0.0119604 | 0.00218232 | 4.20E-08 |
| rs56188862 | 1 | 174189269 | C | T | -0.0157568 | 0.0021747 | 4.30E-13 |
| rs962242 | 1 | 154592140 | C | T | 0.014459 | 0.00253728 | 1.20E-08 |
| rs1156588 | 2 | 58515375 | G | A | -0.015454 | 0.00260325 | 2.90E-09 |
| rs57462170 | 3 | 50239803 | A | G | 0.0191505 | 0.00340563 | 1.90E-08 |
| rs2117137 | 3 | 89525505 | G | A | 0.0129948 | 0.0021557 | 1.70E-09 |
| rs1481012 | 4 | 89039082 | G | A | -0.0262435 | 0.00335608 | 5.30E-15 |
| rs72797284 | 5 | 152031650 | G | A | -0.0171147 | 0.00238353 | 7.00E-13 |
| rs34619 | 5 | 60465365 | A | G | 0.0117117 | 0.0021375 | 4.30E-08 |
| rs7757102 | 6 | 137222671 | G | A | -0.0118039 | 0.00213302 | 3.10E-08 |
| rs2478875 | 6 | 51283110 | G | A | 0.0218943 | 0.00261129 | 5.10E-17 |
| rs2076308 | 6 | 50791640 | C | G | -0.0156639 | 0.00275756 | 1.30E-08 |
| rs149805207 | 6 | 137095269 | G | A | -0.0719337 | 0.0125823 | 1.10E-08 |
| rs11768350 | 7 | 17561651 | C | T | -0.0206574 | 0.00290683 | 1.20E-12 |
| rs4410790 | 7 | 17284577 | C | T | 0.0405506 | 0.00219507 | 3.40E-76 |
| rs713598 | 7 | 141673345 | G | C | 0.0133969 | 0.00215659 | 5.20E-10 |
| rs1078032 | 7 | 17464410 | C | T | 0.0118832 | 0.00214892 | 3.20E-08 |
| rs9648476 | 7 | 39293033 | A | G | 0.0125013 | 0.00218542 | 1.10E-08 |
| rs17656582 | 7 | 17584269 | G | C | 0.0293901 | 0.00461735 | 2.00E-10 |
| rs10273455 | 7 | 17762666 | A | C | -0.0116758 | 0.00213652 | 4.60E-08 |
| rs17685 | 7 | 75616105 | A | G | 0.0230655 | 0.00236195 | 1.60E-22 |
| rs13282783 | 8 | 22088975 | T | C | -0.0135837 | 0.00235432 | 7.90E-09 |
| rs56348300 | 9 | 7054124 | G | C | 0.0158824 | 0.00273191 | 6.10E-09 |
| rs10764990 | 10 | 129152608 | A | G | -0.0121906 | 0.00216898 | 1.90E-08 |
| rs2351187 | 10 | 86850616 | A | G | 0.0129023 | 0.0022823 | 1.60E-08 |
| rs10752269 | 10 | 12692902 | A | G | -0.0128727 | 0.00211975 | 1.30E-09 |
| rs4418728 | 10 | 94839724 | T | G | -0.0117136 | 0.00212761 | 3.70E-08 |
| rs10741694 | 11 | 16286183 | C | T | 0.0150037 | 0.00219355 | 7.90E-12 |
| rs11022752 | 11 | 13307622 | G | A | 0.0133502 | 0.00239391 | 2.50E-08 |
| rs1453548 | 11 | 59192089 | A | T | -0.0133414 | 0.00224973 | 3.00E-09 |
| rs17245213 | 11 | 1679769 | A | G | -0.0146481 | 0.00260905 | 2.00E-08 |
| rs977474 | 12 | 11284772 | T | C | 0.0217813 | 0.00285559 | 2.40E-14 |
| rs2645929 | 13 | 56444529 | G | A | -0.0149842 | 0.0027166 | 3.50E-08 |
| rs6829 | 13 | 111531264 | T | C | -0.0119163 | 0.00216546 | 3.70E-08 |
| rs2783129 | 13 | 80168720 | G | C | -0.0117331 | 0.00213314 | 3.80E-08 |
| rs17576658 | 13 | 100272019 | A | G | -0.0134812 | 0.00245655 | 4.10E-08 |
| rs7999399 | 13 | 89233505 | T | C | 0.0117079 | 0.00213305 | 4.00E-08 |
| rs2472297 | 15 | 75027880 | T | C | 0.0533453 | 0.00240098 | 2.30E-109 |
| rs12591786 | 15 | 60902512 | T | C | -0.0184399 | 0.00294243 | 3.70E-10 |
| rs9937354 | 16 | 53799847 | A | G | -0.0140923 | 0.0021433 | 4.90E-11 |
| rs9302428 | 16 | 24717600 | G | C | 0.0122457 | 0.00220122 | 2.60E-08 |
| rs512404 | 16 | 63031551 | T | G | 0.0150272 | 0.00256164 | 4.50E-09 |
| rs2279844 | 17 | 40819809 | A | G | -0.0119879 | 0.00218318 | 4.00E-08 |
| rs4808193 | 19 | 19410622 | C | T | 0.0151149 | 0.0022472 | 1.70E-11 |
| rs57631352 | 19 | 4338173 | G | A | -0.0131035 | 0.00232117 | 1.70E-08 |
| rs2273447 | 20 | 62900120 | T | A | 0.0174715 | 0.00263421 | 3.30E-11 |
| rs4817505 | 21 | 34343828 | C | T | 0.015068 | 0.0021746 | 4.20E-12 |
| rs132904 | 22 | 41798896 | C | G | 0.0166007 | 0.00255257 | 7.80E-11 |
| rs9624470 | 22 | 24820268 | A | G | 0.0252071 | 0.00215485 | 1.30E-31 |

Abbreviation: SNP, single nucleotide polymorphism; Chr, chromosome; SE, standard error.

**Table S2: Mendelian randomization analysis of tea intake and asthma.**

| SNP | Chr | position | Effect allele | Other allele | Beta | SE | P | F |
| --- | --- | --- | --- | --- | --- | --- | --- | --- |
| rs10752269 | 10 | 12692902 | A | G | -0.000770901 | 0.000646726 | 0.18 | 36.88 |
| rs10764990 | 10 | 129152608 | A | G | 0.000564935 | 0.000662815 | 0.37 | 31.59 |
| rs12591786 | 15 | 60902512 | T | C | 0.00197562 | 0.000897979 | 0.024 | 39.27 |
| rs13282783 | 8 | 22088975 | T | C | 0.00133357 | 0.000721584 | 0.069 | 33.29 |
| rs1453548 | 11 | 59192089 | A | T | -0.000148669 | 0.000686328 | 0.84 | 35.17 |
| rs17245213 | 11 | 1679769 | A | G | 0.00019684 | 0.000802184 | 0.8 | 31.52 |
| rs17576658 | 13 | 100272019 | A | G | 0.00198163 | 0.000758785 | 0.011 | 30.12 |
| rs17656582 | 7 | 17584269 | G | C | -0.00213853 | 0.00143117 | 0.14 | 40.52 |
| rs2076308 | 6 | 50791640 | C | G | 0.000547269 | 0.000836537 | 0.55 | 32.27 |
| rs2117137 | 3 | 89525505 | G | A | 0.000470717 | 0.000658927 | 0.52 | 36.34 |
| rs2351187 | 10 | 86850616 | A | G | 0.000683105 | 0.00070116 | 0.37 | 31.96 |
| rs2478875 | 6 | 51283110 | G | A | 0.000774172 | 0.000801024 | 0.28 | 70.30 |
| rs4418728 | 10 | 94839724 | T | G | 0.0021024 | 0.000650321 | 0.001 | 30.31 |
| rs4817505 | 21 | 34343828 | C | T | 0.000351589 | 0.00066972 | 0.64 | 48.01 |
| rs512404 | 16 | 63031551 | T | G | 0.00012524 | 0.000785131 | 0.82 | 34.41 |
| rs56348300 | 9 | 7054124 | G | C | -0.000375768 | 0.000832692 | 0.66 | 33.80 |
| rs57631352 | 19 | 4338173 | G | A | 0.0016389 | 0.000709304 | 0.021 | 31.87 |
| rs7999399 | 13 | 89233505 | T | C | -0.0010291 | 0.000651964 | 0.14 | 30.13 |
| rs9648476 | 7 | 39293033 | A | G | -0.000183368 | 0.000668274 | 0.87 | 32.72 |

Abbreviation: SNP, single nucleotide polymorphism; Chr, chromosome; SE, standard error.

**Table S3: Mendelian randomization analysis of tea intake and allergic rhinitis.**

| SNP | Chr | position | Effect allele | Other allele | Beta | SE | P | F |
| --- | --- | --- | --- | --- | --- | --- | --- | --- |
| rs10273455 | 7 | 17762666 | A | C | 0.000100037 | 0.00047391 | 0.82 | 29.86 |
| rs10752269 | 10 | 12692902 | A | G | 0.000281425 | 0.000470245 | 0.57 | 36.88 |
| rs10764990 | 10 | 129152608 | A | G | -0.000210095 | 0.000481943 | 0.67 | 31.59 |
| rs12591786 | 15 | 60902512 | T | C | 0.000609645 | 0.000652659 | 0.38 | 39.28 |
| rs13282783 | 8 | 22088975 | T | C | 1.47E-05 | 0.000524488 | 1 | 33.29 |
| rs1453548 | 11 | 59192089 | A | T | -0.00028227 | 0.000499001 | 0.58 | 35.17 |
| rs17245213 | 11 | 1679769 | A | G | 0.00052117 | 0.000583234 | 0.38 | 31.52 |
| rs17576658 | 13 | 100272019 | A | G | 6.23E-05 | 0.000551464 | 0.88 | 30.12 |
| rs17656582 | 7 | 17584269 | G | C | -0.00016658 | 0.00104037 | 0.87 | 40.52 |
| rs2076308 | 6 | 50791640 | C | G | -0.00100945 | 0.000608656 | 0.095 | 32.27 |
| rs2117137 | 3 | 89525505 | G | A | 0.00135931 | 0.000479205 | 0.005 | 36.34 |
| rs2351187 | 10 | 86850616 | A | G | -0.000302642 | 0.000509824 | 0.54 | 31.96 |
| rs2478875 | 6 | 51283110 | G | A | 0.000228794 | 0.000582817 | 0.69 | 70.30 |
| rs4418728 | 10 | 94839724 | T | G | -0.000109433 | 0.000472859 | 0.83 | 30.31 |
| rs4817505 | 21 | 34343828 | C | T | 0.000271196 | 0.00048661 | 0.59 | 48.01 |
| rs512404 | 16 | 63031551 | T | G | -0.000741165 | 0.000570704 | 0.2 | 34.41 |
| rs56348300 | 9 | 7054124 | G | C | -0.000726523 | 0.000605416 | 0.22 | 33.80 |
| rs57631352 | 19 | 4338173 | G | A | 0.000465285 | 0.000515558 | 0.34 | 31.87 |
| rs6829 | 13 | 111531264 | T | C | -0.000408606 | 0.000484694 | 0.38 | 30.28 |
| rs7999399 | 13 | 89233505 | T | C | -0.000538876 | 0.000473829 | 0.25 | 30.13 |
| rs9648476 | 7 | 39293033 | A | G | -0.000384572 | 0.000485793 | 0.47 | 32.72 |

Abbreviation: SNP, single nucleotide polymorphism; Chr, chromosome; SE, standard error.

**Table S4: Mendelian randomization analysis of tea intake and chronic cough.**

| SNP | Chr | position | Effect allele | Other allele | Beta | SE | P | F |
| --- | --- | --- | --- | --- | --- | --- | --- | --- |
| rs10273455 | 7 | 17762666 | A | C | 0.00143759 | 0.00145829 | 0.32 | 29.86 |
| rs10741694 | 11 | 16286183 | C | T | 0.000952434 | 0.00149901 | 0.53 | 46.78 |
| rs10752269 | 10 | 12692902 | A | G | -0.00173255 | 0.00144872 | 0.23 | 36.88 |
| rs10764990 | 10 | 129152608 | A | G | 0.000875786 | 0.00148193 | 0.55 | 31.59 |
| rs1078032 | 7 | 17464410 | C | T | -0.000963151 | 0.00146699 | 0.51 | 30.58 |
| rs11022752 | 11 | 13307622 | G | A | 0.00211629 | 0.00163613 | 0.2 | 31.10 |
| rs11164870 | 1 | 93552187 | G | C | -0.000643962 | 0.00149502 | 0.67 | 30.04 |
| rs1156588 | 2 | 58515375 | G | A | 0.00381556 | 0.00177477 | 0.032 | 35.24 |
| rs11587444 | 1 | 150722844 | G | A | 0.00168487 | 0.00148891 | 0.26 | 41.79 |
| rs11768350 | 7 | 17561651 | C | T | -0.0023956 | 0.00198456 | 0.23 | 50.50= |
| rs12591786 | 15 | 60902512 | T | C | 0.00349363 | 0.00201284 | 0.083 | 39.27 |
| rs13282783 | 8 | 22088975 | T | C | 0.000725785 | 0.00160755 | 0.65 | 33.29 |
| rs132904 | 22 | 41798896 | C | G | -0.00353745 | 0.00174763 | 0.043 | 42.30 |
| rs1453548 | 11 | 59192089 | A | T | -0.00182025 | 0.00153508 | 0.24 | 35.17 |
| rs1481012 | 4 | 89039082 | G | A | -0.00233678 | 0.0022888 | 0.31 | 61.15 |
| rs17245213 | 11 | 1679769 | A | G | 0.000272622 | 0.0017821 | 0.88 | 31.52 |
| rs17576658 | 13 | 100272019 | A | G | 0.00112886 | 0.00167711 | 0.5 | 30.12 |
| rs17656582 | 7 | 17584269 | G | C | 0.00266552 | 0.00315924 | 0.4 | 40.52 |
| rs2117137 | 3 | 89525505 | G | A | -0.000165965 | 0.0014723 | 0.91 | 36.34 |
| rs2351187 | 10 | 86850616 | A | G | -0.000273284 | 0.00155057 | 0.86 | 31.96 |
| rs4817505 | 21 | 34343828 | C | T | -0.000112359 | 0.00148914 | 0.94 | 48.01 |
| rs512404 | 16 | 63031551 | T | G | -0.00238939 | 0.00174677 | 0.17 | 34.41 |
| rs56348300 | 9 | 7054124 | G | C | -0.000758997 | 0.00186612 | 0.68 | 33.80 |
| rs57631352 | 19 | 4338173 | G | A | 0.00295366 | 0.00158218 | 0.062 | 31.87 |
| rs6829 | 13 | 111531264 | T | C | 0.000445654 | 0.00147644 | 0.76 | 30.28 |
| rs7999399 | 13 | 89233505 | T | C | -0.000202534 | 0.00145714 | 0.89 | 30.13 |
| rs9648476 | 7 | 39293033 | A | G | -0.00047656 | 0.00149364 | 0.75 | 32.72 |

Abbreviation: SNP, single nucleotide polymorphism; Chr, chromosome; SE, standard error.

**Table S5: Mendelian randomization analysis of tea intake and eczema.**

| SNP | Chr | position | Effect allele | Other allele | Beta | SE | P | F |
| --- | --- | --- | --- | --- | --- | --- | --- | --- |
| rs10273455 | 7 | 17762666 | A | C | 0.00019 | 0.000322 | 0.51 | 29.86 |
| rs10741694 | 11 | 16286183 | C | T | 8.07E-05 | 0.000331 | 0.81 | 46.78 |
| rs10752269 | 10 | 12692902 | A | G | 6.68E-05 | 0.00032 | 0.83 | 36.88 |
| rs10764990 | 10 | 1.29E+08 | A | G | -5.86E-05 | 0.000328 | 0.89 | 31.59 |
| rs1078032 | 7 | 17464410 | C | T | 0.000451 | 0.000325 | 0.17 | 30.58 |
| rs11022752 | 11 | 13307622 | G | A | 0.000619 | 0.00036 | 0.081 | 31.10 |
| rs1156588 | 2 | 58515375 | G | A | -0.00068 | 0.000393 | 0.085 | 35.24 |
| rs11768350 | 7 | 17561651 | C | T | -0.00065 | 0.000443 | 0.14 | 50.50 |
| rs12591786 | 15 | 60902512 | T | C | -0.0005 | 0.000444 | 0.25 | 39.27 |
| rs13282783 | 8 | 22088975 | T | C | -2.38E-05 | 0.000357 | 0.95 | 33.29 |
| rs132904 | 22 | 41798896 | C | G | -0.00048 | 0.000384 | 0.21 | 42.30 |
| rs1453548 | 11 | 59192089 | A | T | 0.000505 | 0.000339 | 0.13 | 35.17 |
| rs1481012 | 4 | 89039082 | G | A | -0.00032 | 0.000509 | 0.57 | 61.18 |
| rs17245213 | 11 | 1679769 | A | G | 0.000662 | 0.000397 | 0.084 | 31.52 |
| rs17576658 | 13 | 1E+08 | A | G | -0.00052 | 0.000375 | 0.16 | 30.12 |
| rs17656582 | 7 | 17584269 | G | C | 0.001319 | 0.000708 | 0.064 | 40.52 |
| rs17685 | 7 | 75616105 | A | G | -0.00018 | 0.000356 | 0.6 | 95.36 |
| rs2076308 | 6 | 50791640 | C | G | -0.00045 | 0.000414 | 0.26 | 32.27 |
| rs2117137 | 3 | 89525505 | G | A | 0.000307 | 0.000326 | 0.34 | 36.34 |
| rs2273447 | 20 | 62900120 | T | A | -0.00058 | 0.000397 | 0.15 | 43.99 |
| rs2279844 | 17 | 40819809 | A | G | 0.000456 | 0.00033 | 0.17 | 30.15 |
| rs2351187 | 10 | 86850616 | A | G | 8.15E-05 | 0.000347 | 0.81 | 31.96 |
| rs2472297 | 15 | 75027880 | T | C | 0.000868 | 0.00037 | 0.019 | 493.64 |
| rs2478875 | 6 | 51283110 | G | A | 0.000397 | 0.000396 | 0.34 | 70.30 |
| rs2645929 | 13 | 56444529 | G | A | 4.41E-05 | 0.000407 | 0.93 | 30.42 |
| rs2783129 | 13 | 80168720 | G | C | 0.000463 | 0.000324 | 0.13 | 30.25 |
| rs34619 | 5 | 60465365 | A | G | 0.00055 | 0.000323 | 0.098 | 30.02 |
| rs4410790 | 7 | 17284577 | C | T | 0.000128 | 0.000331 | 0.71 | 341.27 |
| rs4418728 | 10 | 94839724 | T | G | -0.00044 | 0.000322 | 0.18 | 30.31 |
| rs4808193 | 19 | 19410622 | C | T | 0.000203 | 0.000338 | 0.56 | 45.24 |
| rs4817505 | 21 | 34343828 | C | T | -0.00047 | 0.000331 | 0.14 | 48.01 |
| rs512404 | 16 | 63031551 | T | G | 0.000448 | 0.000388 | 0.26 | 34.41 |
| rs56188862 | 1 | 1.74E+08 | C | T | 0.000398 | 0.000329 | 0.26 | 52.50 |
| rs56348300 | 9 | 7054124 | G | C | 0.000348 | 0.000412 | 0.39 | 33.80 |
| rs57462170 | 3 | 50239803 | A | G | 0.000134 | 0.000515 | 0.81 | 31.62 |
| rs57631352 | 19 | 4338173 | G | A | 0.000671 | 0.000351 | 0.057 | 31.87 |
| rs6829 | 13 | 1.12E+08 | T | C | 9.40E-06 | 0.00033 | 0.99 | 30.28 |
| rs713598 | 7 | 1.42E+08 | G | C | 0.00015 | 0.000325 | 0.64 | 38.59 |
| rs72797284 | 5 | 1.52E+08 | G | A | -0.00058 | 0.000363 | 0.12 | 51.56 |
| rs7999399 | 13 | 89233505 | T | C | -0.00011 | 0.000322 | 0.77 | 30.13 |
| rs9302428 | 16 | 24717600 | G | C | -0.00018 | 0.000333 | 0.61 | 30.95 |
| rs962242 | 1 | 1.55E+08 | C | T | 0.00019 | 0.000386 | 0.62 | 32.47 |
| rs9648476 | 7 | 39293033 | A | G | -2.22E-06 | 0.00033 | 0.97 | 32.72 |
| rs977474 | 12 | 11284772 | T | C | 0.000838 | 0.000428 | 0.051 | 58.18 |
| rs9937354 | 16 | 53799847 | A | G | 0.000489 | 0.000324 | 0.13 | 43.23 |

Abbreviation: SNP, single nucleotide polymorphism; Chr, chromosome; SE, standard error.

**Table S6: Mendelian randomization analysis of tea intake and atopic dermatitis.**

| SNP | Chr | position | Effect allele | Other allele | Beta | SE | P | F |
| --- | --- | --- | --- | --- | --- | --- | --- | --- |
| rs10273455 | 7 | 17762666 | A | C | -0.0039 | 0.0102 | 0.698 | 29.86 |
| rs10741694 | 11 | 16286183 | C | T | 1.00E-04 | 0.0115 | 0.991 | 46.78 |
| rs10752269 | 10 | 12692902 | A | G | -0.0097 | 0.0104 | 0.353 | 36.88 |
| rs10764990 | 10 | 129152608 | A | G | -0.003 | 0.0102 | 0.771 | 31.59 |
| rs11768350 | 7 | 17561651 | C | T | -0.0141 | 0.014 | 0.314 | 50.50 |
| rs12591786 | 15 | 60902512 | T | C | 0.0277 | 0.0154 | 0.071 | 39.27 |
| rs13282783 | 8 | 22088975 | T | C | 0.0138 | 0.0105 | 0.191 | 33.29 |
| rs1453548 | 11 | 59192089 | A | T | -0.017 | 0.0108 | 0.115 | 35.17 |
| rs1481012 | 4 | 89039082 | G | A | 0.0323 | 0.0177 | 0.069 | 61.15 |
| rs149805207 | 6 | 137095269 | G | A | 0.0385 | 0.0449 | 0.391 | 32.68 |
| rs17245213 | 11 | 1680039 | A | G | 9.00E-04 | 0.0129 | 0.946 | 31.52 |
| rs17576658 | 13 | 100272019 | A | G | 0.0063 | 0.0123 | 0.609 | 30.12 |
| rs17656582 | 7 | 17584269 | G | C | 0.0449 | 0.0217 | 0.039 | 40.52 |
| rs17685 | 7 | 75616105 | A | G | -0.0071 | 0.0108 | 0.509 | 95.36 |
| rs2076308 | 6 | 50791640 | C | G | -0.0017 | 0.0121 | 0.888 | 32.27 |
| rs2117137 | 3 | 89587262 | G | A | 0.0065 | 0.0102 | 0.522 | 36.34 |
| rs2273447 | 20 | 62900120 | T | A | 0.0195 | 0.0137 | 0.154 | 43.99 |
| rs2279844 | 17 | 40819809 | A | G | 0.0096 | 0.0104 | 0.356 | 30.15 |
| rs2351187 | 10 | 86850616 | A | G | -0.0137 | 0.0111 | 0.220 | 31.96 |
| rs4817505 | 21 | 34343828 | C | T | -0.0246 | 0.0104 | 0.018 | 48.01 |
| rs512404 | 16 | 63031551 | T | G | -0.0174 | 0.0123 | 0.156 | 34.41 |
| rs56348300 | 9 | 7054124 | G | C | 0.0096 | 0.0143 | 0.502 | 33.80 |
| rs57631352 | 19 | 4338173 | G | A | 0.0136 | 0.0108 | 0.208 | 31.87 |
| rs7999399 | 13 | 89233505 | T | C | -0.0239 | 0.0103 | 0.020 | 30.13 |
| rs962242 | 1 | 154592140 | C | T | 0.0029 | 0.0125 | 0.814 | 32.47 |
| rs9648476 | 7 | 39293033 | A | G | -0.011 | 0.0105 | 0.296 | 32.72 |
| rs977474 | 12 | 11284772 | T | C | 0.0178 | 0.0167 | 0.287 | 58.18 |

Abbreviation: SNP, single nucleotide polymorphism; Chr, chromosome; SE, standard error.
